# Editorial: Obsessive-Compulsive Related Disorders: Towards an Advancement of the Knowledge of These Internalizing Disorders

**DOI:** 10.3389/fpsyg.2022.849610

**Published:** 2022-02-08

**Authors:** Yura Loscalzo, Marco Giannini, Kenneth G. Rice

**Affiliations:** ^1^Department of Health Sciences, School of Psychology, University of Florence, Florence, Italy; ^2^Center for the Study of Stress, Trauma, and Resilience, Department of Counseling and Psychological Services, Georgia State University, Atlanta, GA, United States

**Keywords:** OCD, Studyholism, body dysmorphic disorder, excoriation, trichotillomania, hoarding disorder, study addiction, behavioral addiction

This Research Topic arose from the editors' interest in Obsessive-Compulsive related Disorders and a new potential clinical disorder associated with problematic overstudying, which has been conceptualized as a behavioral addiction (Atroszko et al., 2015) or an OCD-related disorder (Loscalzo and Giannini, 2017, 2018). Loscalzo and Giannini (2017), in line with Kardefelt-Winther's (2015) recommendations, considered alternative criteria to addiction to identify the authentic manifestation of problematic overstudying. This seemed critical considering the increasing tendency to introduce new behavioral addictions. The year when the DSM-5 (American Psychiatric Association, 2013) was published saw a peak of 2,563 papers about behavioral addictions (Billieux et al., 2015). The DSM-5 (American Psychiatric Association, 2013) included Gambling Disorder in the addictive behaviors section and Internet Gaming Disorder in the section related to Emerging Measure and Models. This may have legitimized the study of new behavioral addictions. However, we reached a point where almost every common behavior has been labeled as a potential behavioral addiction, such as tanning (Kourosh et al., 2010) and fortune-telling (Grall-Bronnec et al., 2015). Therefore, Reinarman and Granfield (2014) suggested that scholars have become “addicted to addiction” (p. 1). More importantly, as denounced by Billieux et al. (2015), creating these diagnoses generally follows a three-step approach that is atheoretical and confirmatory. First, following an anecdotal observation, researchers consider the excessive behavior as an addictive behavior (*a priori* approach). Next, they develop a screening instrument referring to the core symptoms of substance addictions (confirmatory approach). Finally, they carry on studies to unearth those risk factors that are usually associated with substance addictions and that, by analogy, they suppose characterize the new behavioral addiction (confirmatory approach). However, there are some critical problems and issues with these steps. First, an anecdotal observation should not be sufficient for proposing a new behavioral addiction. Second, Billieux et al. (2015) observed that developing items based on the criteria of addictive disorders is not simple for all the addictions components, especially as concerns tolerance, which is one of the key addiction features (Shaffer et al., 2004). Moreover, there are several instruments proposed to evaluate these new excessive behaviors. Besides being based on the core components of addiction, these instruments, in some cases, come dangerously close to duplicating previous instruments (Griffiths et al., 2016). Finally, Billieux et al. (2015) pointed out that, by using instruments based on the addiction framework, it is obvious that the researchers will find the expected relationships with the risk factors typical of addictions. Moreover, they underscored that the addiction model leads to a lack of specificity and theoretically sound models that are able to specify the particular factors involved in each problematic and excessive behavior.

In light of these issues, Starcevic (2016) suggested taking a step back to recall that a few years ago, there was a similar excess regarding obsessive-compulsive spectrum disorders; it seems prudent to pay attention to history and avoid repeating the same mistakes. He believes that disorders characterized by repetitive and problematic behaviors and poor impulse control might be conceptualized using the addiction framework, but there is no evidence that other theoretical approaches are inferior. Hence, he stressed that we still have to understand whether specific behaviors are better conceptualized as impulse control disorder, obsessive-compulsive disorder, addiction, or other. Surprisingly, the situation has not much improved, despite Starcevic's (2016) cautions regarding a prior excess of diagnoses concerning OCD-related disorders. Separate literature searches (using the OneSearch database) conducted at the beginning of January 2022, restricted to peer-reviewed papers, and using the keywords “behavioral addiction” and “Obsessive Compulsive related Disorder” yielded, respectively, 151,070 and 60,536 papers. The number of publications about behavioral addictions was more than double the number of OCD-related papers. Hence, the present Research Topic aimed to prompt scholars contributing to the advancement of knowledge about OCD-related disorders, pointing to balance the wide attention currently given to the addiction/externalizing framework, and hence *trying to retrieve the main essence of scientific research*: looking for the real nature of phenomena while avoiding aprioristic and confirmatory approaches.

Besides Loscalzo and Giannini's paper about Studyholism (or obsession toward study), which provided some evidence about its conceptualization as an OCD-related disorder (or, more generally, as an internalizing disorder), there are other interesting studies about hoarding, trichotillomania, and skin picking disorder. He et al. investigated the relationship between hoarding and compulsive buying, highlighting a positive correlation between the two conditions. Grant et al., analyzing both trichotillomania and skin picking, showed a lack of relationship between past-year fat/sugar dietary intake and these disorders' severity, while they found an association between nutrition and impulsivity and compulsivity (as transdiagnostic factors). Next, Kłosowska et al. showed that traumatic life events (e.g., emotional neglect, sexual abuse, and bodily threat) might contribute to the development of skin picking as a regulation strategy and that dissociative symptoms partially explained the relationship, for both the automatic/unconscious and focused skin picking style. In line with these findings, Gallinat et al. highlighted that most subjects experience a loss of control, trance, and positive feelings during pathological skin-picking episodes. However, these episodes are often preceded by boredom, bodily tension, and strong negative feelings, and they are usually followed by shame, guilt, and anger. Among the main findings, they also showed that skin picking severity is positively associated with body image disturbances and low self-esteem. Finally, Lochner et al. suggested that factors other than emotion dysregulation should be analyzed as potential contributors to trichotillomania, based on their findings that patients have higher rates of childhood trauma, perceived stress, and emotion dysregulation than healthy controls. However, they found no association between emotion dysregulation and the severity of hair-pulling. Moreover, the association between perceived stress and emotion dysregulation was not specific to trichotillomania; it was also evident in the healthy group.

In conclusion, the interesting findings included in the current Research Topic provide ample justification for further, and similarly creative, attention on OCD-related disorders. Moreover, analyzing excessive behaviors using an OCD-related perspective—and not only an addiction one—might help reveal the real nature of understudied (or new potential) clinical diagnoses.

## Author Contributions

YL wrote the draft of the manuscript. MG and KGR critically revised its content. All authors contributed to the article and approved the submitted version.

## Conflict of Interest

The authors declare that the research was conducted in the absence of any commercial or financial relationships that could be construed as a potential conflict of interest.

## Publisher's Note

All claims expressed in this article are solely those of the authors and do not necessarily represent those of their affiliated organizations, or those of the publisher, the editors and the reviewers. Any product that may be evaluated in this article, or claim that may be made by its manufacturer, is not guaranteed or endorsed by the publisher.
